# Measurement precision enhancement of surface plasmon resonance based angular scanning detection using deep learning

**DOI:** 10.1038/s41598-022-06065-2

**Published:** 2022-02-08

**Authors:** Kitsada Thadson, Suvicha Sasivimolkul, Phitsini Suvarnaphaet, Sarinporn Visitsattapongse, Suejit Pechprasarn

**Affiliations:** 1 Department of Biomedical Engineering, School of Engineering, King Mongkut’s Institute of Technology Ladkrabang, Bangkok, 10520 Thailand; 2 College of Biomedical Engineering, Rangsit University, Pathum Thani, 12000 Thailand

**Keywords:** Optical techniques, Imaging and sensing, Applied optics, Optical sensors, Optical physics, Nanophotonics and plasmonics

## Abstract

Angular scanning-based surface plasmon resonance measurement has been utilized in label-free sensing applications. However, the measurement accuracy and precision of the surface plasmon resonance measurements rely on an accurate measurement of the plasmonic angle. Several methods have been proposed and reported in the literature to measure the plasmonic angle, including polynomial curve fitting, image processing, and image averaging. For intensity detection, the precision limit of the SPR is around 10^–5^ RIU to 10^–6^ RIU. Here, we propose a deep learning-based method to locate the plasmonic angle to enhance plasmonic angle detection without needing sophisticated post-processing, optical instrumentation, and polynomial curve fitting methods. The proposed deep learning has been developed based on a simple convolutional neural network architecture and trained using simulated reflectance spectra with shot noise and speckle noise added to generalize the training dataset. The proposed network has been validated in an experimental setup measuring air and nitrogen gas refractive indices at different concentrations. The measurement precision recovered from the experimental reflectance images is 4.23 × 10^–6^ RIU for the proposed artificial intelligence-based method compared to 7.03 × 10^–6^ RIU for the cubic polynomial curve fitting and 5.59 × 10^–6^ RIU for 2-dimensional contour fitting using Horner's method.

## Introduction

Surface plasmon resonance (SPR) is a phenomenon that occurs at the surface of noble metals^1^, such as gold and silver. The resonating electron on a metal surface can be excited by light illumination matching the resonant frequency, causing a resonant oscillation and generating a surface wave propagating along the metal surface called a plasmonic wave or surface plasmon polaritons. The light to electron energy coupling leads to an energy loss appearing as an intensity dip in reflectance spectra; a dark band called SPR dip or plasmonic dip. The surface plasmon coupling condition is sensitive to the external environment in contact with the plasmonic metal's surface. Therefore, it has been widely utilized as a label-free, non-invasive, and real-time sensor and has gained interest in many research fields, such as SPR-based sensing^2–5^, SPR-based microscopy^6^, voltage sensing^7,8^, biomolecular interaction analysis^9,10^, environment monitoring^11,12^ and medical diagnosis^13–15^.

The reflectance spectra can be measured using a camera sensor or a linear photodiode array^12^ that transforms photons into electrons. However, for the SPR measurement, the camera captures an image of the SPR reflectance dip in which the optical intensity is typically low and usually in a shot-noise dominant measurement^16,17^. Therefore, the measurement precision depends on the accuracy of a plasmonic dip position measurement under the noise constraint. Several minimum reflectance dip determination methods have been proposed and developed, including polynomial curve fitting^18,19^, cross correlation^20^, and centroid determination^21^.

Convolutional neural network (CNN) is a well-known type of deep learning widely employed in image recognition because of its ability to analyze and recognize a spatial data pattern^22^. The network has been utilized in many fields and applications, such as medical imaging^23^, image classification^24,25^, image regression^26,27^, phase retrieval^28^, and image enhancement^29^.

Here, we propose a deep learning-based method for automatically and accurately locating the plasmonic dip position in real-time to enhance precision in the plasmonic measurement of surface plasmon resonance-based angular scanning detection using the CNN architecture. We also demonstrate that simulated data can be employed to train the neural network. First, the simulated reflectance spectra with added shot-noise and speckle noise were prepared to mimic images from the experimental setup generalizing the simulated dataset for the CNN training. The proposed trained network was later evaluated in an experimental setup measuring refractive index change in real-time to quantify the measurement precision enhancement compared to conventional methods, including one-dimension and two-dimensional curve fitting methods. To the best of the authors' knowledge, the proposed CNN method to enhance the accuracy in SPR measurement has never been reported before in the literature.

## Materials and methods

### Surface plasmon resonance optical alignment

**Figure 1:**
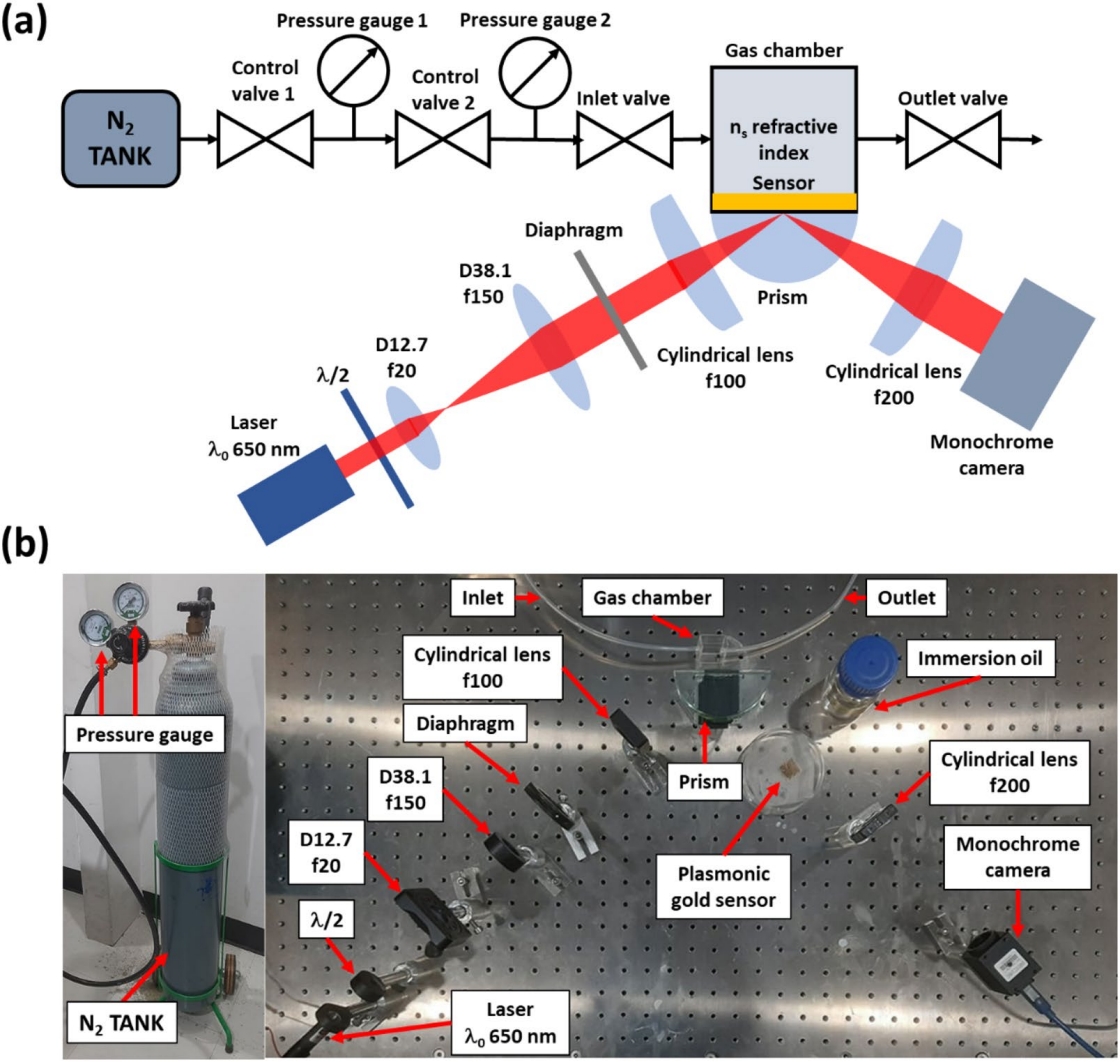
Kretschmann-based SPR experiment equipped with an acrylic gas chamber and nitrogen gas control unit (**a**) schematic diagram of the SPR system, (**b**) photo the experimental setup.

An SPR angular interrogation-based Kretschmann configuration was aligned to demonstrate the capability of the proposed CNN method compared with cubic polynomial curve fitting and Horner's method. The setup consisted of two main parts: a gas flow-control system for varying the refractive index of the SPR sensing region and an optical system, as depicted in Fig. 1.

The gas flow-control system equipped with pressure gauges was employed to control nitrogen gas pressure in the sensing region (gas chamber), providing an independent measurement to cross-validate and approximate the refractive indices of the gas inside the chamber. The gas system consisted of a nitrogen gas tank, a gas regulator for pressure control, an inlet valve, an 18 × 18 × 18 mm^3^ plastic gas chamber, and an outlet valve.

The optical detection system captured the reflectance spectrum using a CMOS camera (MT9P006, Huatengvision). The camera was monochromatic with a bit-depth of 8 bits, 2592 pixels × 1944 pixels, pixels size 2.2 μm × 2.2 μm, the camera well depth of 4796 electrons per pixel, and quantum efficiency of 61% at the incident wavelength λ of 650 nm. The plasmonic layer consisted of a 45 nm plasmonic gold with a 2 nm chromium adhesion layer on a standard BK7 microscope coverslip (No. 0, Sigma-Aldrich) prepared by electron beam sputtering. The gold plasmonic sensor was mounted on an 80 mm diameter semicylindrical glass prism (80 mm prism, Scitrader) using a microscope matching oil (MOIL-30, Olympus). For SPR excitation, the system consisted of a 7 mW laser diode 650 nm (L650P007, Thorlabs), a half waveplate (WPH10E-633, Thorlabs); an achromatic plano-convex doublet lens with a diameter of 12.7 mm and focal length of 20 mm (LA1074-A-N-BK7, Thorlabs) and an achromatic plano-convex doublet lens with a diameter of 38.1 mm and focal length of 150 mm (LA1388-A-N-BK7, Thorlabs) for beam expansion. The magnified beam then passed through an iris diaphragm (D255, Thorlabs), reducing the beam diameter to 20 mm. The beam is then focused using a 1-inch plano-convex cylindrical lens with a focal length of 100 mm (LJ1567L2-A-N-BK7). Finally, a plano-convex cylindrical lens expands the reflected beam with a focal length of 200 nm (LJ1309L1-A-N-BK7). The coverage incident angle of the sensor can be calculated from the optical system to 5.73 × 10^–4^ degrees per pixel or 0.00001 in sinθ_0_ unit, similar to the angular resolution employed in the literature^30^. The 5.73 × 10^–4^ degrees per pixel or 0.00001 in sinθ_0_ unit were calculated from the demagnified camera sensor size of 5.7 mm using two cylindrical lenses, providing a demagnification factor of 2 when projecting back to the f100 cylindrical lens, as shown in Fig. 1. The demagnified camera pixel can cover a specific range of incident angles, which can be calculated using numerical aperture (NA) relationship sin(tan^−1^(D/2f)) corresponding to the NA of 0.014, where D is the camera size. The 2NA can then be divided by the total number of pixels of 2592 pixels along with the angular space leading to the angular resolution of 0.00001 in sinθ_0_.

SPR active research groups have widely adopted the camera-based SPR dip measurement for various applications^31,32^, although measurement accuracy and precision can be improved by employing a linear diode array^33^. Here, the paper aims to demonstrate that the measurement precision can be improved by increasing the efficacy of data utilization using artificial intelligence compared to the other conventional methods with no need for additional optical instrument modification.

### Experimental procedure

The experimental procedure consisted of (1) opening the inlet and outlet valves for airflow through the gas chamber for SPR detection at the air refractive index stage, (2) flowing the nitrogen gas to replace the air in the gas chamber, (3) closing the outlet valve for keeping the nitrogen gas of 5 pressure levels of 5 psi, 40 psi, 110 psi, 130 psi, and 150 psi, respectively, (4) closing the inlet-controlled valve when the pressure raised to the stabilized pressure. The plasmonic dip was then measured as a video file capturing at an 8-fps camera framerate. At the stable pressure levels, 100 video frames for each pressure level were employed as a testing dataset to evaluate the performance of three plasmonic angle measurement methods. The experiment was carried out when the vibration isolation system was switched off and deflated the optical table. Note that the gas pressure range is the maximum pressure that the plastic chamber can withstand before the gas leaks out, and the range is within the detection requirements for gas sensing reported in the literature^34,35^.

### Calibration procedure and method to recover sample's refractive index

The plasmonic angles recovered for the air case from each method were calibrated to the same initial value of sinθ_sp_ of 0.6783, corresponding to the air refractive index of 1.000276^36^. The sinθ_sp_ of 0.6783 was obtained from the theoretical value computed using Fresnel equations and the transfer matrix approach for the 45 nm gold and the 2 nm chromium layer on a BK7 glass substrate and the incident wavelength of 650 nm computed using the chromium and gold refractive indices reported by Johnson and Christy^37^. Then the change in the plasmonic angle Δsinθ_sp_ due to the nitrogen pressures can be then determined based on the number of pixels that the plasmonic dip moves compared to the air case.

### Nitrogen gas pressures

The gas refractive index can be related to its concentration, proportional to the gas pressure. The experimental setup was in a condition of a constant temperature and volume. A gas refractive index can be estimated as gas pressure based on the empirical equation and Boyle's relationship. For nitrogen and air compound in a constant volume chamber, the gas pressure and its corresponding gas refractive index were extracted from the experimental results reported in Wong et al.^38^ and fitted using a linear function as expressed in Eq. (1). Note that the coefficient of determination R^2^ of 0.9999 and the root mean square error (RMSE) of 1.0087 × 10^−6^ RIU.

1 $$n_{s} = 1.892395105 \times 10^{ - 5} P + 1.000277923$$

where, *n*_*s*_ is the gas refractive index, and P is the tank pressure in the Pa unit.

### Data analysis procedure

The video recorded file from the experiment described in the earlier section was analyzed using the following three methods:

#### Cubic polynomial curve fitting

**Figure 2:**
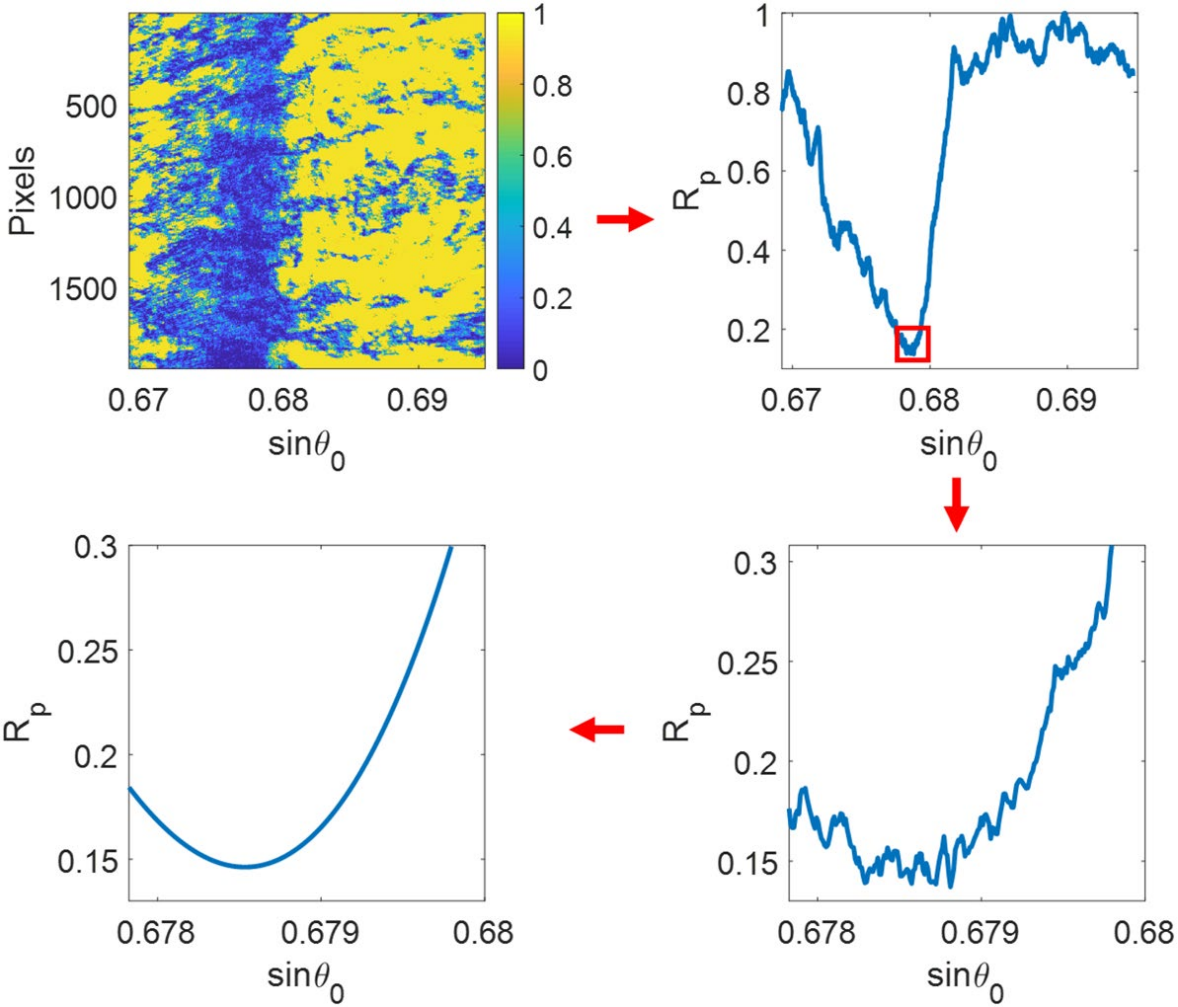
Steps in the cubic polynomial curve fitting-based method for determining the minimum position of SPR reflectance dip.

Figure 2 shows the process flow to determine the plasmonic angle from a recorded camera frame. First, the line-scan reflectance was prepared by averaging all the rows in the camera frame and applying a cubic polynomial curve fitting^39^ through the minimum reflectance for the averaged SPR dip. It will be shown later in the “Results” section that the accuracy of the curve fitting method depends on the number of data points included in the polynomial curve fitting.

#### Horner's curve fitting method

**Figure 3:**
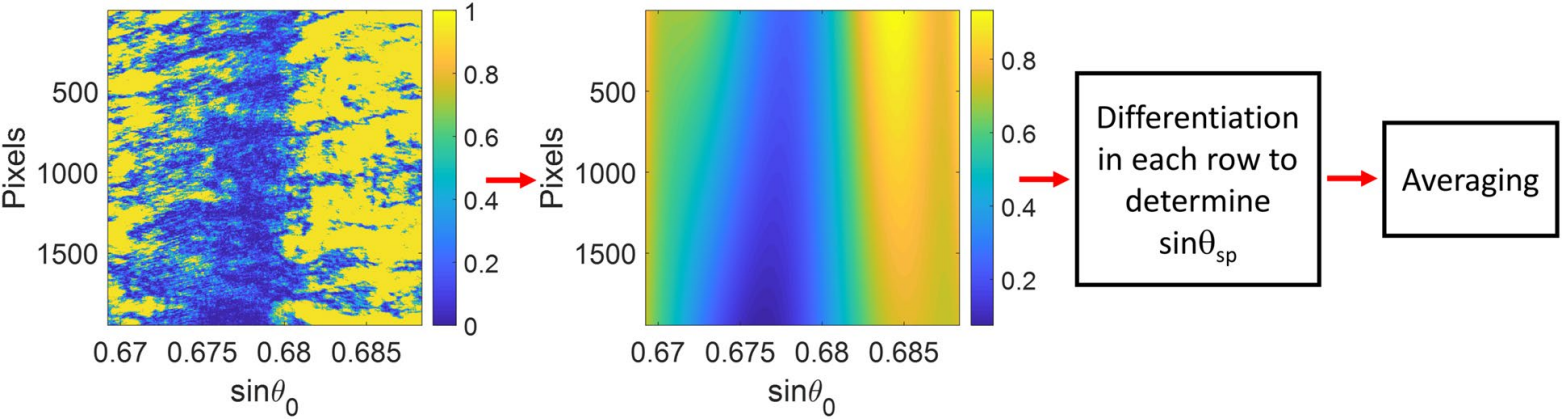
Steps in Horner's curve fitting for SPR dip estimation.

Horner's curve fitting method is a well-known algorithm for 2-dimensional polynomial surface fitting. This method was employed to calculate a fitting surface of plasmonic dip image data, as depicted in Fig. 3 and expressed in Eq. (2). First, a line-scan reflectance was evaluated from the curve fitting contour by considering all the rows. Then, the 2D function with polynomial coefficients allowed us to accurately obtain the sinθ_sp_ for each row using function differentiation; these sinθ_sp_ values were averaged to determine the sinθ_sp_ for a camera frame.

It is crucial to point out that the plasmonic dips are usually asymmetrical because of the loss and coupling mechanism^40^. Several asymmetrical SPR dip curve fitting techniques claim the performance enhancement, such as the sigmoid-asymmetric fitting algorithm^18^ and poles-zeros-based fitting function^41^. Horner's curve fitting nature allows the cross-polynomial terms between x and y to form an asymmetric fitting, as depicted in Fig. 3. As a result, the method should provide better data usage of the asymmetrical fitting and noise averaging than the symmetrical one-dimensional curve fitting explained earlier.

2 $$\begin{aligned} f(x,y) & = p_{1} x^{n} y^{m} + p_{2} x^{(n - 1)} y^{m} + \cdots + p_{n + 1} y^{m} + \cdots \\ & \quad p_{n + 2} x^{n} y^{m - 1} + p_{n + 3} x^{(n - 1)} y^{m - 1} + \cdots + p_{2(n + 1)} y^{(m - 1)} + \cdots \\ & \quad \cdots \\ & \quad p_{m(n + 1) + 1} x^{n} + p_{m(n + 1) + 2} x^{(n - 1)} + \cdots + p_{(n + 1)(m + 1)} \\ \end{aligned}$$

where, *p*_*n*_ are the polynomial coefficients. n is the number of orders of x, which is the sinθ_0_. m is the number of orders of y, which is the row number of the camera frame, and in this study, m is 1. In other words, linear response along the camera rows.

#### CNN based method

The CNN predicted the plasmonic angle position in the pixel, which later converted to the plasmonic angle sinθ_sp_. Table 1 shows the proposed neural network architecture for the image regression problem in this study. The network consisted of an input image size of 70 pixels × 2592 pixels and four additional levels. Each level consisted of a 9 × 9 × 32 convolutional layer, a batch normalization layer, and a rectified linear unit (ReLU) layer. The last layer was a 1 × 1 fully connected layer and a mean-absolute-error regression layer to estimate the pixel number of the minimum reflectance with four additional decimal points, which was equivalent to 0.00000001 in sinθ_0_ unit and 4.20 × 10^–8^ RIU. Note that the minimum RIU corresponded to the current state-of-the-art RIU measurement precision of metasurfaces^42,43^ and phase-sensitive SPR measurement^44,45^.

**Table 1:** The proposed convolutional neural network architecture.

Layers	Activations	Learnable variables	Descriptions
Image input	70 × 2592 × 1	–	70 × 2592 × 1 images
Convolution		Weights 9 × 9 × 1 × 32 Bias 1 × 1 × 32	1 stride, 1 padding
Batch normalization		Offset 1 × 1 × 32 Scale 1 × 1 × 32	–
ReLU		–	–
Convolution		Weights 9 × 9 × 1 × 32 Bias 1 × 1 × 32	1 stride, 1 padding
Batch normalization		Offset 1 × 1 × 32 Scale 1 × 1 × 32	–
ReLU		–	–
Convolution	70 × 2592 × 32	Weights 9 × 9 × 1 × 32 Bias 1 × 1 × 32	1 stride, 1 padding
Batch normalization		Offset 1 × 1 × 32 Scale 1 × 1 × 32	–
ReLU		–	–
Convolution		Weights 9 × 9 × 1 × 32 Bias 1 × 1 × 32	1 stride, 1 padding
Batch normalization		Offset 1 × 1 × 32 Scale 1 × 1 × 32	–
ReLU		–	–
Fully connected	1 × 1 × 1	Weights 1 × 5,806,080 Bias 1 × 1	1 fully connected
Regression output	–	–	Mean absolute error

The training options consisted of adaptive momentum estimation (Adam) optimizer^46^, batch size of 64 images, and 0.0001 initial learning rate. The CNN training was under a single graphic processor unit (GPU) NVIDIA TITAN RTX with 16 GB RAM. Note that the 64 images were the maximum number of images under the 16 GB RAM environment. The effect of the number batch size will be discussed in the “Results” section.

The network was trained with 1000 epochs to ensure that the root-mean-square validation error (RMSE) reached the convergence of RMSE of 10^–4^ pixels, which was the same level as the decimal precision.

It is essential to point out that the 70 input rows were much lower than the 1944 rows in the camera frame. It will be shown later that this is not due to the memory limitation in the GPU, but the higher number of rows can degrade the accuracy.

### Training dataset preparation for CNN training

**Figure 4:**
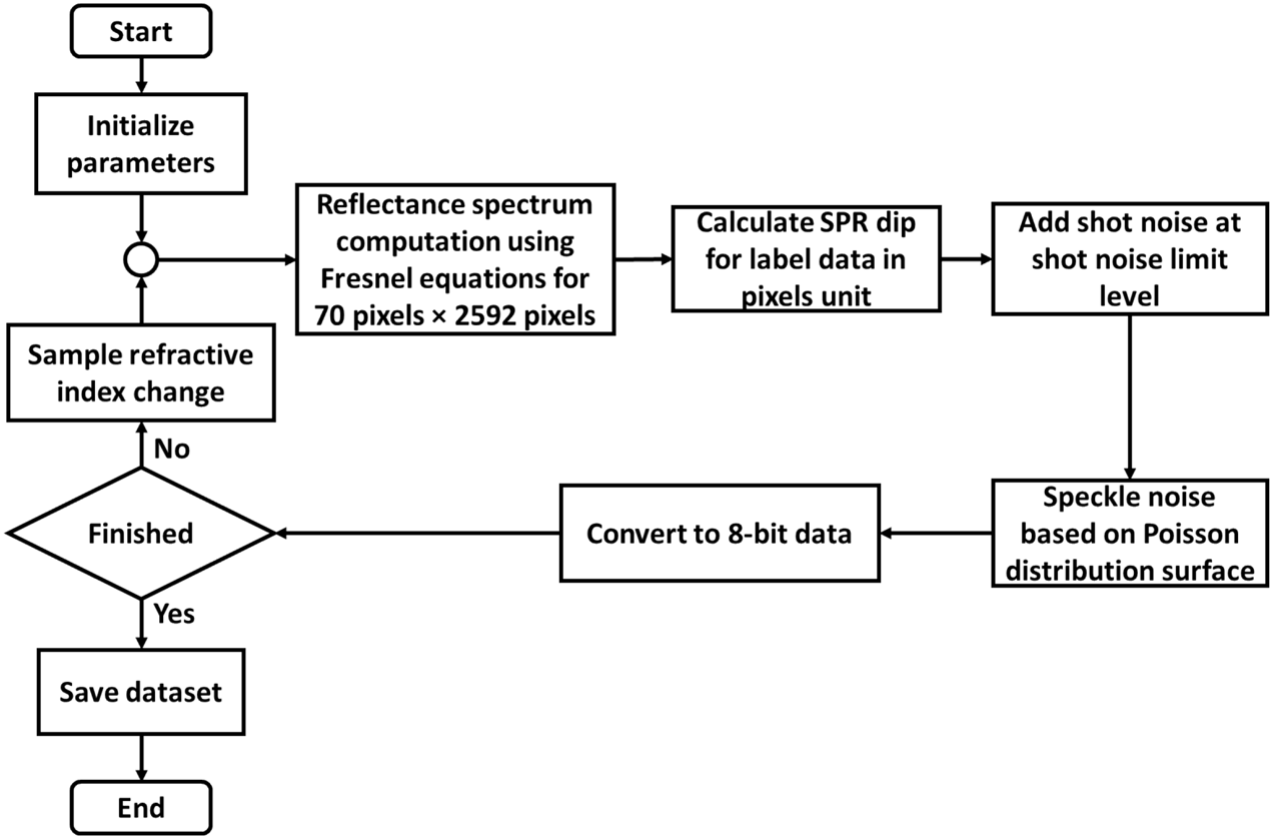
Flowchart of a simulated dataset process.

The training dataset was simulated camera frames computed using the Fresnel equations and the transfer matrix method^47^. The simulation process is as described and shown in Fig. 4. Firstly, each camera frame had 70 pixels in a row and the same columns as raw data of 2592 pixels. The control parameters for simulated reflectance consisted of a 45 nm gold thickness-based sensor with a 650 nm wavelength source mimicking the experimental setup. The simulation then varied the refractive index in the sensing region to form 64 camera frames with 8-bit precision covering the sample refractive index of 1.00–1.05 with the RIU step size of 0.00078 RIU increment.

**Figure 5:**
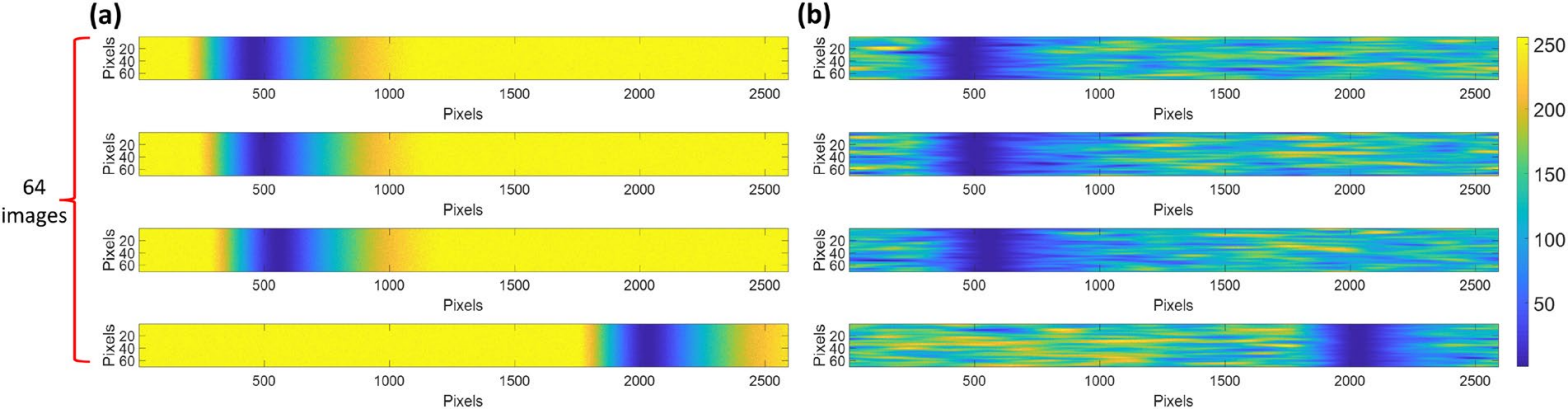
(**a**) 64 camera frames corresponding to different sample refractive indices with shot-noise, and (**b**) shot-noise and speckle pattern added camera frames.

The shot and speckle noises were added to the simulated images to generalize and mimic the experimental responses; experimental data obtained in an SPR experiment is usually shot noise dominant due to low optical intensity at the plasmonic dip^30^. The shot noise was randomly added with the noise amplitude proportional to $\sqrt {R_{p} }$, where $R_{p}$ is the reflectance simulated using Fresnel equations and the transfer matrix approach, as depicted in Fig. 5a and followed by adding a random speckle-noise pattern, as shown in Fig. 5b. The random speckle-noise patterns were generated using the random Gaussian process method proposed by Byun et al*.*^48^. The process can be summarized as (1) generating a digital randomized array of 0 and 1 for 70 pixels × 2592 pixels. (2) The digital randomized array was then convoluted to a Gaussian distribution function with a mean of 0 pixels and a standard deviation of 100 pixels to generate a speckle pattern, which was (3) then normalized the speckle pattern to 0 to 1 range and added to the shot-noise added images. These simulated camera frames were then applied in the CNN training.

#### Testing dataset for the CNN based method

**Figure 6:**
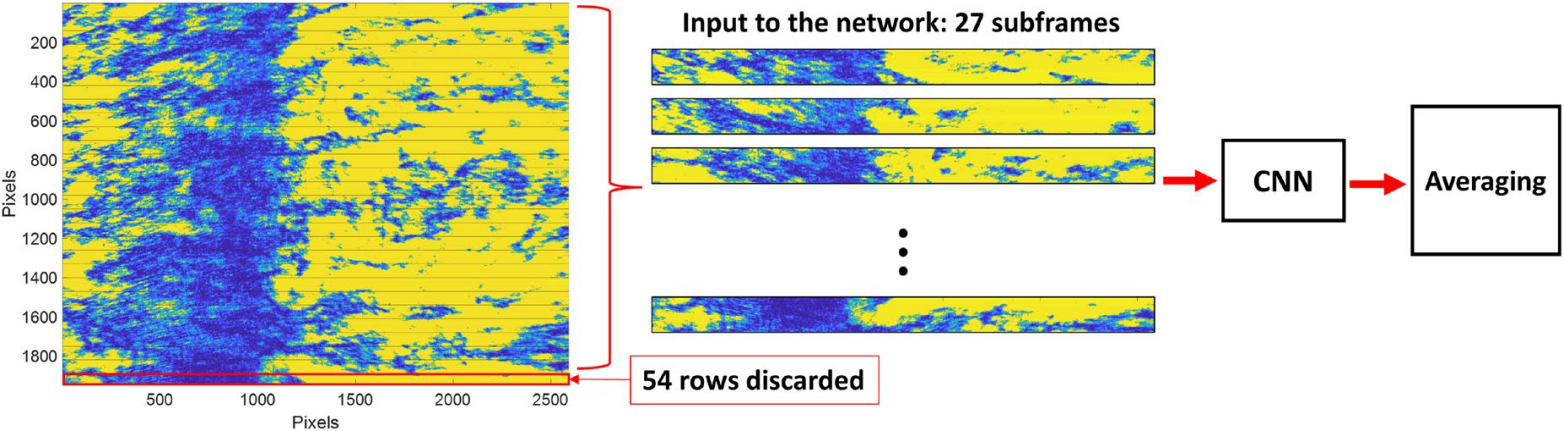
The DL testing process analyzing the experimental data.

The testing dataset was the experimental data obtained from the experimental procedure explained in the “Experimental procedure” section. The raw data from the camera was recorded in a video format that captured a range of refractive index changes from air to different nitrogen pressures of 5 psi, 40 psi, 110 psi, 130 psi, and 150 psi. Each camera frame was then separated into subframes with an identical size of 70 pixels × 2592 pixels with no overlapping rows, as depicted in Fig. 6. Thus, there were 27 subframes from each video frame, and the bottom 54 rows were excluded from the CNN analysis. The 27 subframes were then analyzed using the trained CNN network, providing 27 regression outputs in the pixel unit corresponding to the minimum plasmonic reflectance captured in each subframe. The averaged pixel value of the 27 regression outputs was then evaluated, converted, and calibrated to sinθ_sp_ using the calibration process explained in the calibration section.

### Quantitative parameters

Sensitivity (S) is defined as the sensor plasmonic angular response $\Delta \sin_{{{\text{sp}}}}$ over the change in the refractive index in the sensing chamber $\Delta {\text{n}}_{{\text{s}}}$, as expressed in Eq. (3).

3 $$S = \frac{\partial Signal}{{\partial Measurand}} = \frac{{\Delta sin_{sp} }}{{\Delta n_{s} }}$$

Measurement standard deviation ($\sigma$) represents the lowest quantity that the sensor can measure, which is of ${\text{sin}}_{{{\text{sp}}}}$ values recovered from 100 recovered ${\text{sin}}_{{{\text{sp}}}}$ values for each gas pressure and the three measurement methods.

## Results and discussion

**Figure 7:**
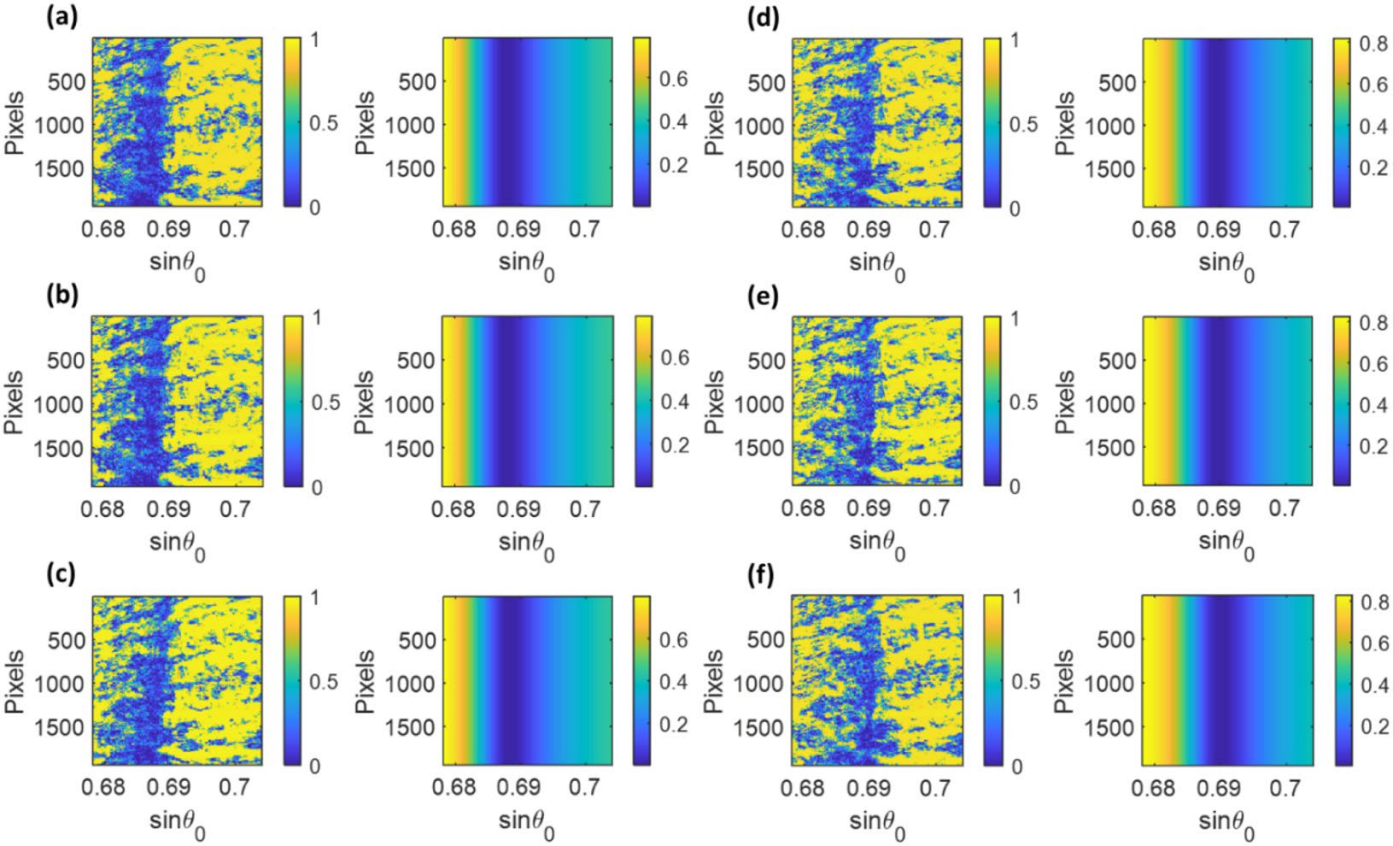
Experimental camera frames (left) and simulated reflectance spectra (right) showing the plasmonic dip positions for 6 different pressures: (**a**) air case, (**b**) 5 psi, (**c**) 40 psi, (**d**) 110 psi, (**e**) 130 psi, and (**f**) 150 psi.

The experiment was calibrated at the initial air stage at 23 °C standard room temperature and pressure; Fig. 7a shows the corresponding SPR angular response. The N_2_ pressure was then increased to the five pressure levels of 5 psi, 40 psi, 110 psi, 130 psi, and 150 psi; the SPR angular responses for each pressure level were shown in Fig. 7b–f, respectively. Note that the equivalent reflectance spectra shown on the right side of Fig. 7a–f were simulated the Fresnel equation and the transfer matrix approach explained earlier with the sample refractive indices of 1.000276 for the refractive index of air^31^ and 1.000373 1.001035, 1.002360, 1.002738 and 1.003117 for the other five N_2_ pressure levels calculated using Eq. (1).

The first 100 frames for the air case were analyzed using the three methods working out the corresponding plasmonic angles from the three methods and calibrating them to the sinθ_sp_ of 0.6783 for the theoretical air refractive index 1.000276^36^.

### Cubic polynomial method

For the curve fitting method, the number of data points around the minimum reflectance dip plays a crucial role in the accuracy of the plasmonic dip. The number of data points included in the cubic polynomial fitting varied from 41 to 401 pixels to demonstrate the point. The minimum value of this fitted curve is an approximated SPR dip position in pixels unit.

**Figure 8:**
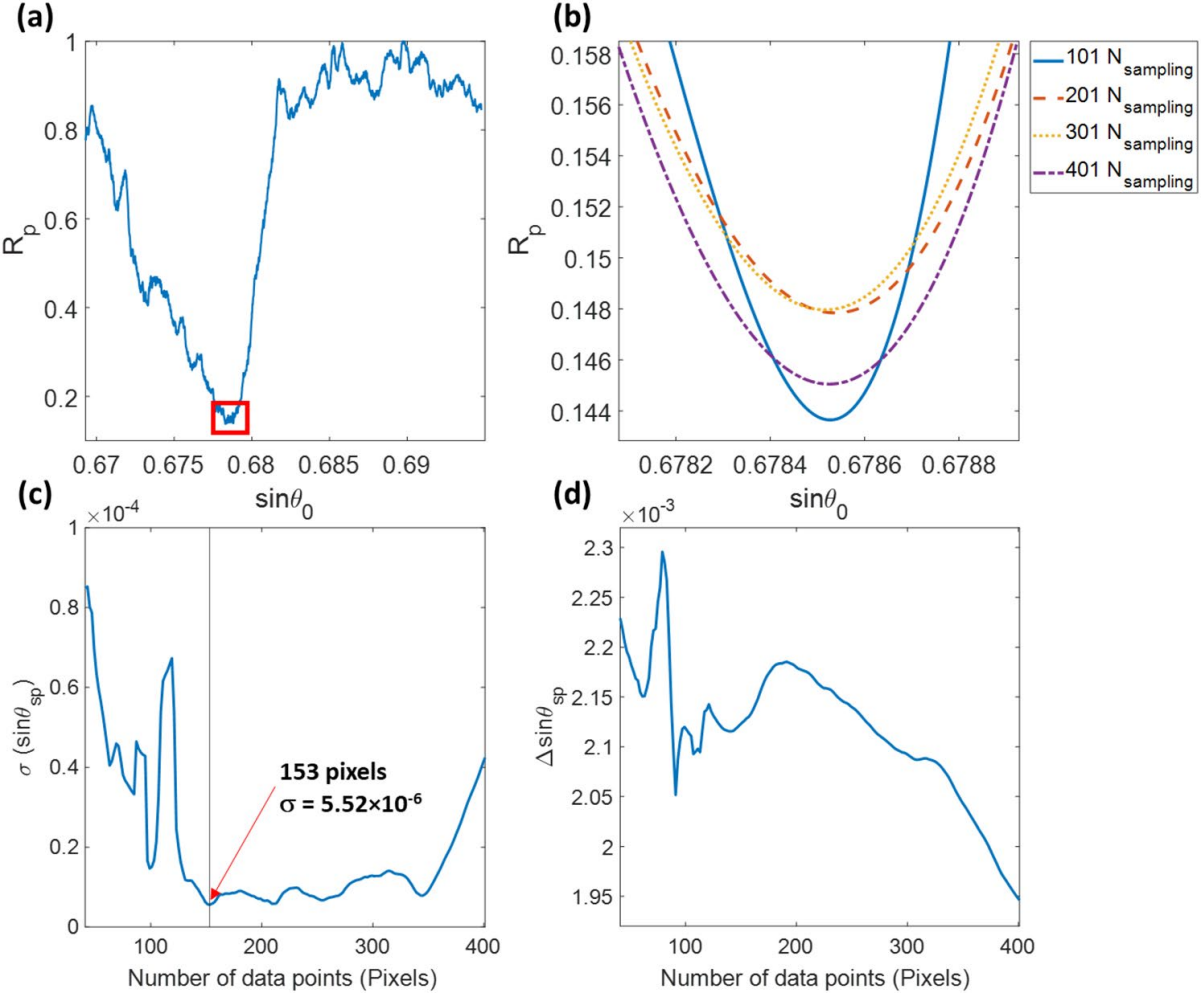
(**a**) Average line-scan calculated from 1944 rows of the first frame of the air case, (**b**) cubic polynomial fitting curves for data points of 101 (solid blue curve), 201 (dashed red curve). 301 (dashed yellow curve). 401 (dashed purple curve), (**c**) σ of 100 recovered sinθ_sp_ values for each data point width, and (**d**) Δsinθ_sp_ between the air and 150 psi cases for each data point width.

The first camera frame of the experimental data for the air case shown in Fig. 7a was averaged to a 1D line scan, as shown in Fig. 8a. Then the different amount of data points around the minimum dip was included in cubic spline polynomial curve fitting, as shown in Fig. 8b. Next, the corresponding sinθ_sp_ for each data point widths were evaluated and stored. Finally, the same analysis was applied to the other 99 air case images and the σ of the 100 recovered sinθ_sp_ values for each data point width, as shown in Fig. 8c. For example, the number of data points of 153 had the lowest σ of 5.52 × 10^–6^ in sinθ_sp_, corresponding to 3.16 × 10^–4^ degrees and 7.03 × 10^–6^ RIU. The number of data points included in the curve fitting also affect the sensitivity; here, we applied the same data processing to the data obtained for the pressure level of 150 psi and determined the averaged sinθ_sp_ for the same data point widths. The Δsinθ_sp_ values between the two cases for the analyzed data points are shown in Fig. 8d. The higher number of data points degraded the sensitivity performance for the polynomial curve fitting method. The average sinθ_sp_ and σ values for all the pressure levels recovered using the polynomial curve fitting are summarized in Table 2 compared to the other methods.

**Table 2:** Mean and standard deviation values of plasmonic dip from three methods in sinθ unit.

	Plasmonic dip in sinθ_sp_					
	Cubic polynomial		Horner's method		CNN	
	μ	σ	μ	σ	μ	σ
Air		5.52 × 10^–6^		4.39 × 10^–6^		3.32 × 10^–6^
N2 at 5 psi	0.67843	5.05 × 10^–6^	0.67842	4.70 × 10^–6^	0.67841	3.54 × 10^–6^
N2 at 40 psi	0.67881	5.93 × 10^–6^	0.67894	4.22 × 10^–6^	0.67883	3.69 × 10^–6^
N2 at 110 psi	0.67986	5.86 × 10^–6^	0.67986	4.55 × 10^–6^	0.67969	3.77 × 10^–6^
N2 at 130 psi	0.68013	5.33 × 10^–6^	0.68014	4.49 × 10^–6^	0.67996	4.06 × 10^–6^
N2 at 150 psi	0.68046	5.84 × 10^–6^	0.68047	4.74 × 10^–6^	0.68042	3.85 × 10^–6^

### Horner's method

**Figure 9:**
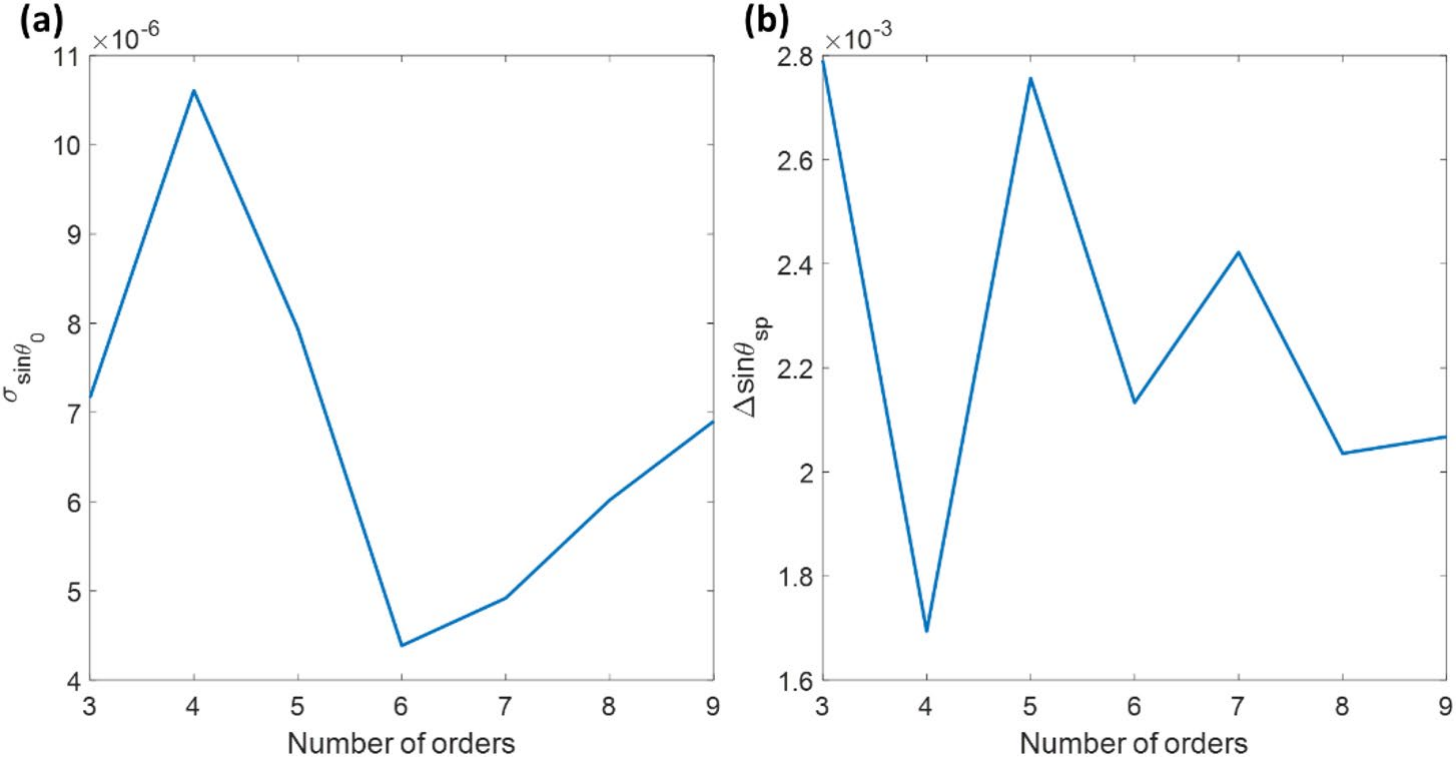
(**a**) σ of 100 recovered sinθ_sp_ values for each data point width, and (**b**) Δsinθ_sp_ between the air and 150 psi cases using Horner's curve fitting method with varying the n polynomial order from 3 to 9.

The same experimental test dataset was analyzed using Horner's method for surface fitting. First, the images were cropped to 1944 pixels × 1944 pixels, where the 648 rightmost columns were discarded from the computation to center the plasmonic dip since they did not contain reflectance dip information, as shown in Fig. 7a. There were two polynomial degrees in Eq. (2): the parameters n for the x-axis (sinθ_0_) and m for the y-axis (camera's row). The m parameter was fixed at 1, mimicking the consistent plasmonic dip line-scans in all the rows. The n parameter was then varied from the 3rd to the 9th polynomial order to determine the effects of the polynomial on the recovered measurement values by evaluating σ for the 100 frames of the air case and Δsinθ_sp_ between the air, and 150 psi cases, as shown in Fig. 9a,b. The lowest σ of 4.39 × 10^–6^ in sinθ_sp_, corresponding to 2.34 × 10^–4^ degrees and 5.59 × 10^–6^ RIU, was at the n of 6 orders, which was lower than the cubic polynomial fitting. Thus, Horner's method can utilize the image data more effectively using the surface fitting, considering the relationship between rows and columns. Horner's method based on sixth-order polynomial was then employed to analyze the other pressure levels and summarized in Table 2, comparing to the other measurement methods.

### CNN based method

**Figure 10:**
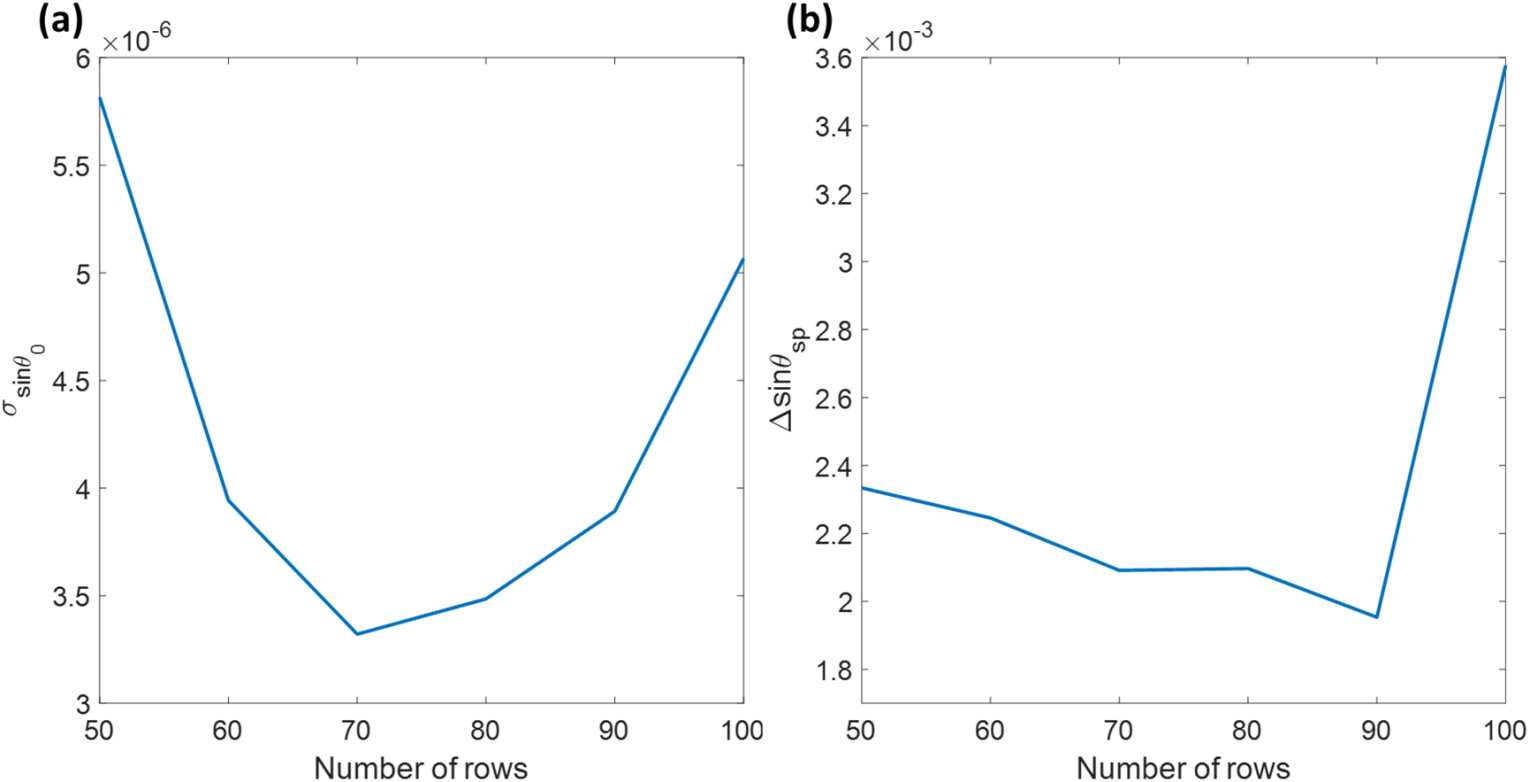
(**a**) σ of 100 recovered sinθ_sp_ values, and (**b**) Δsinθ_sp_ between the air and 150 psi cases using CNN networks with various input rows ranging from 50 to 100 rows.

Multiple networks with varying input row numbers were trained for 50 rows to 100 rows optimizing for the lowest σ value. All the networks and training functions were the same except the number of input rows. The network with 100 rows was the last configuration to be trained under the GPU before exceeding the GPU memory capacity. Figure 10a shows the $\sigma$ values for the 100 experimental frames from the air backing experiment. The network output was in pixels, later converted to sinθ_sp_ by the calibration process described in the “Materials and methods” section. Figure 10b shows the Δsinθ_sp_ between the air and 150 psi cases analyzed using the networks. The number of input rows did affect the sensitivity much when it was less than 100 rows; moreover, the higher row numbers did not produce a higher measurement accuracy. It is possibly due to the relative size of noise artifacts in the experimental results. The lowest σ of 3.32 × 10^–6^ in sinθ_sp_, corresponding to 1.90 × 10^–4^ degrees and 4.23 × 10^–6^ RIU, was at the input row number of 70. Like the first two SPR dip recovery methods, all the other gas concentrations were then analyzed using the CNN-based network with 70 pixels × 2592 pixels input image size.

**Figure 11:**
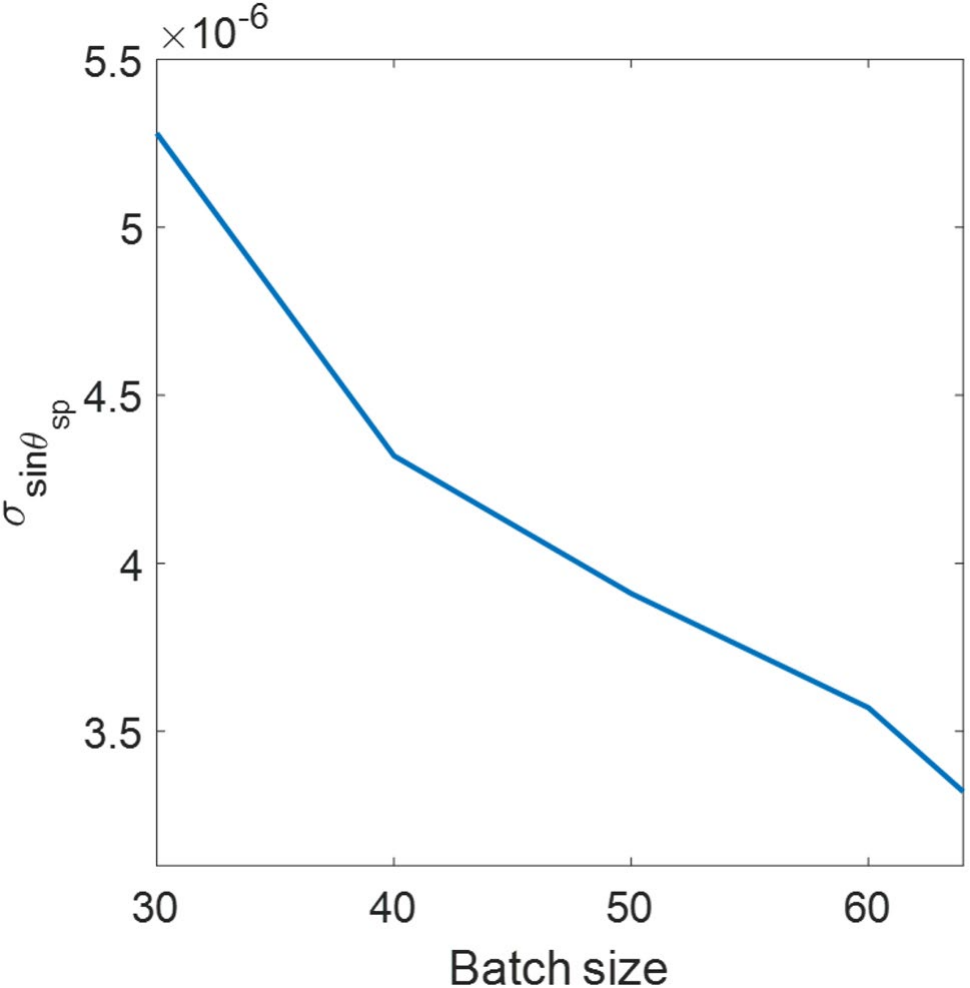
Shows σ of 100 recovered sinθ_sp_ values for the air case analyzed using CNN networks trained with a varying batch size ranging from 30 to 64 images.

The results in Fig. 10 were analyzed using the batch size of 64 images. Figure 11 shows the effect of networks trained using different batch sizes ranging from 20 to 64 images. The trained networks were then applied to analyze the 100 experimental frames for the air case to evaluate the standard deviation and the mean value. Note that the 64 images were the maximum limit for the computer memory consisting of 64 images of 8-bit grey-scale images with the size of 70 × 2592 pixels. Therefore, a larger batch size can provide higher accuracy for the same number of epochs of 1000. In contrast, the smaller batch size needs a higher epoch. In other words, it requires a longer training time to achieve the same accuracy. Therefore the batch size of 64 images was applied in the analysis for the following section.

### Performance comparison of the three methods

Table 2 summarizes the recovered mean values and standard derivations of all the three mentioned plasmonic angle recovery methods in sinθ_sp_. The σ values of the three methods indicated that the cubic spline had the worse performance with an averaged σ of 5.59 × 10^–6^; followed by Horner's curve fitting method with an averaged σ of 4.51 × 10^–6^, and the best-performed method was the CNN with its average σ of 3.71 × 10^–6^. Therefore, the CNN can improve the σ value by 66% and 32% compared to the cubic polynomial curve fitting and Horner's method. The stability in the σ measurement tells us about the reproducibility of experiments. The three methods had a similar reproducibility performance regardless of the measurement methods and the gas concentrations.

**Figure 12:**
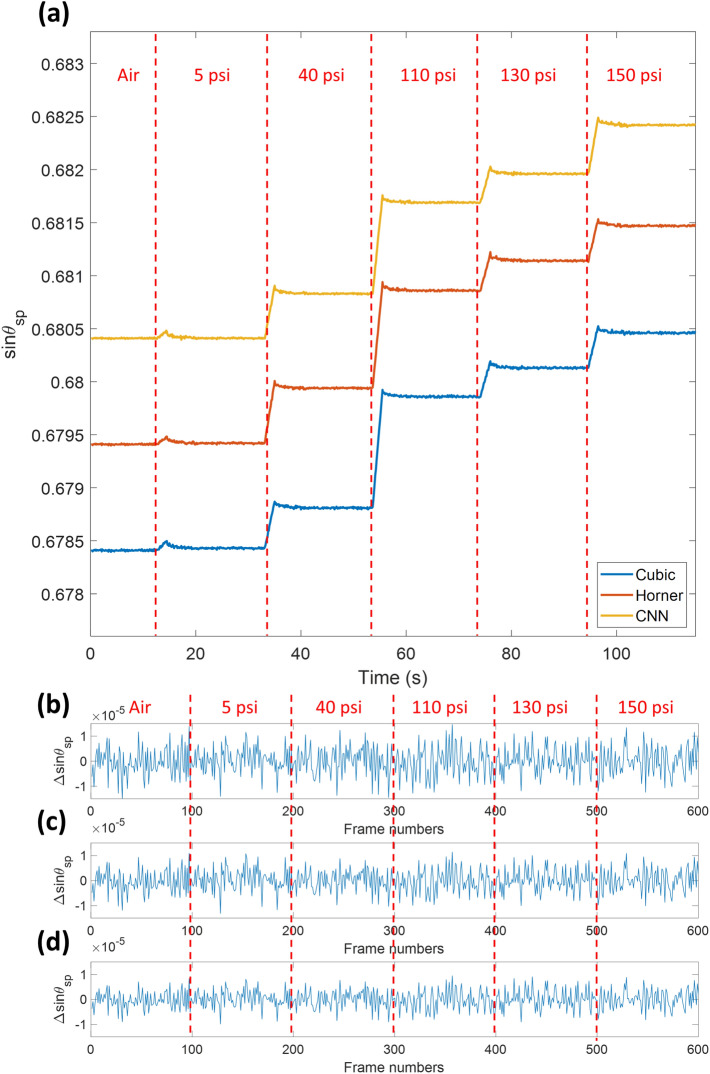
(**a**) Sensorgram of three methods for the five N_2_ pressure levels with 0.005 offset for each sensorgram, (**b**) Δsinθ_sp_ noise levels in the cubic polynomial method, (**c**) Δsinθ_sp_ noise levels in Horner's method, and (**d**) Δsinθ_sp_ noise levels in the CNN method.

Figure 12 shows the sensorgram for the air case and the 5 N_2_ pressures and summarized in Table 2 for 100 frames of stabilized responses for each case. Student *t* tests were then applied to statistically analyze and compare the recovered sinθ_sp_ values for the three plasmonic angle measurement methods. For the cubic polynomial curve fitting and Horner's method, the t and the p values were 0.1872 and 0.8517, respectively. The cubic polynomial curve fitting and the CNN method had the t value of 0.3990 and the p-value of 0.6903. In addition, Horner's method and the CNN method had the t value of 0.2271 and the p-value of 0.8206. The three measurement methods were not significantly different, which indicated that the sinθ_sp_ values recovered from the three methods agreed with each other. The statistical analysis shows that CNN has the narrowest distribution, followed by Horner's method and the cubic polynomial curve fitting.

The sinθ_sp_ in Table 2 can then be converted to the corresponding refractive index change compared to the theoretical values calculated from Fresnel equations and the transfer matrix approach, as reported in Table 3. For precision and sensitivity demanding applications, including single-molecule detection, DNA and virus detection, scientists and engineers would need to increase the measurement precision of their sensor as much as possible. In addition, it is challenging to enhance the measurement performance, especially when the system operates near its measurement precision limit^49^. Here we demonstrate one of the ways to use the measurement data and the artificial intelligence to learn and relate the measurement noise to cancel out the noise and improve the measurement accuracy with no need for additional instrumentation and sophisticated equipment for any existing SPR setup. Only the software upgrade is needed, and also the software upgrade and the CNN training can be done as a background operating when the SPR system is in use.

**Table 3:** Mean and standard deviation values of different refractive indices from three methods.

	Equation (1)	Recovered refractive index					
		Cubic polynomial		Horner's method		CNN	
		μ	σ	μ	σ	μ	σ
Air			7.03 × 10^–6^		5.59 × 10^–6^		4.23 × 10^–6^
N2 at 5 psi	1.000373	1.000396	6.43 × 10^–6^	1.000369	5.98 × 10^–6^	1.000367	4.51 × 10^–6^
N2 at 40 psi	1.001035	1.000938	7.55 × 10^–6^	1.001128	5.37 × 10^–6^	1.000967	4.70 × 10^–6^
N2 at 110 psi	1.002360	1.002469	7.46 × 10^–6^	1.002479	5.80 × 10^–6^	1.002222	4.81 × 10^–6^
N2 at 130 psi	1.002738	1.002867	6.79 × 10^–6^	1.002880	5.73 × 10^–6^	1.002622	5.18 × 10^–6^
N2 at 150 psi	1.003117	1.003352	7.44 × 10^–6^	1.003362	6.04 × 10^–6^	1.003291	4.91 × 10^–6^

Table 3 shows the recovered refractive index inside the sensing chamber, and the σ values in the RIU unit, and the expected refractive index calculated using the pressure reading from the second pressure gauge and Eq. (1) to validate the recovered refractive indices. Again, the pressure gauge reading and the SPR measurements agree well.

The current state-of-the-art measurement precision for angular scanning SPR ranges from 10^–5^ RIU^50,51^ to 10^–6^ RIU^52^ for angular interrogation measurements. Therefore, the $\sigma$ value of the CNN agrees with the reported measurement precision values in the literature. In addition, several optical techniques^53^ have been employed to enhance the limit involving a more demanding optical instrumentation, for example, an optical interferometer^54^, a spatial light modulator-based illumination^55^, metasurfaces^56,57^, and nanowires^58^.

Here, we have demonstrated that the proposed CNN method can enhance and utilize the data by finding the relationship between pixels to cancel the noise out. In other words, deep learning provides more efficient data utilization than the other two conventional methods without generating any new information. This study's three plasmonic angle measurement methods had a similar sensitivity performance of 53.75 degrees/RIU, 53.83 degrees/RIU, and 53.96 degrees/RIU calculated using Eq. (3) for cubic polynomial method, Horner's method, and CNN method, respectively. The sensitivity error was less than 0.4%. It, however, cannot reach the ultralow measurement precision like in the phase-sensitive techniques since the phase information was not in the experimental data. Developing a phase retrieval deep learning-based for angular interrogation measurement; higher frequency components, such as sharper plasmonic phase transition, are artificially added to the data by deep learning. However, the artificially added information by deep learning is questionable.

## Conclusion

The proposed deep learning for SPR measurement precision enhancement of surface plasmon resonance-based angular scanning detection has been developed. The convolutional neural network was utilized to approximate plasmonic angle position in pixel and later converted to corresponding plasmonic angle. The designed CNN network architecture was designed for this purpose. The simulated dataset was simulated reflectance spectra of the p-polarized incident wave at 650 nm wavelength, computed using the Fresnel equation and the transfer matrix for network training. The paper has also demonstrated that simulated data can be employed for deep learning neural network training; the trained network was later tested with experimental data. The experimental setup was developed, integrating an optical system and a gas flow-control system for experimental data collection measuring six refractive index levels controlled by gas pressure. The experimental data were analyzed using two conventional curve fitting methods compared to the proposed CNN network. The three measurement methods show similar sensitivity and reproducibility responses.

Furthermore, the refractive indices recovered using the SPR measurements agreed well with the refractive indices converted from recorded gas pressures. The measurement standard deviations were 4.23 × 10^–6^ RIU for the proposed CNN compared to 7.03 × 10^–6^ RIU for the cubic polynomial curve fitting and 5.59 × 10^–6^ RIU for Horner's method corresponding to 66% and 32% enhancement. The CNN operates by identifying the relationship between every pixel in the input frame of the CNN, leading to more efficient usage of existing data than the cubic polynomial curve fitting and Horner's method without creating an artificial profile or higher frequency components.
